# The Association Between Ideal Cardiovascular Health and Health-Related Quality of Life in Adults: A Population-Based Cross-Sectional Study

**DOI:** 10.3389/ijph.2021.592043

**Published:** 2021-03-19

**Authors:** Xueyan Wu, Xiaotian Liu, Wei Liao, Ning Kang, Shengxiang Sang, Tanko Abdulai, Miaomiao Niu, Yaling He, Zhihan Zhai, Mingming Pan, Chongjian Wang, Yuqian Li

**Affiliations:** ^1^ Department of Epidemiology and Biostatistics, College of Public Health, Zhengzhou University, Zhengzhou, China; ^2^ Department of Clinical Pharmacology, School of Pharmaceutical Science, Zhengzhou University, Zhengzhou, China

**Keywords:** ideal cardiovascular health, health-related quality of life, EQ-5D-5L, rural population, Ideal health behaviors, Ideal health factors

## Abstract

**Objectives:** The study aimed to explore the association between Ideal cardiovascular health (ICH) and health-related quality of life (HRQoL) using the European Quality of Life Five Dimension Five Level Scale (EQ-5D-5L) among rural population.

**Methods:** This study included 20,683 participants aged 18–79 years from “the Henan Rural Cohort study”. Generalized linear and Tobit regression models were employed to explore the associations of ICH with EQ-5D-5L utility scores and visual analogue scale (VAS) scores, respectively.

**Results:** The mean EQ-5D-5L utility scores and VAS scores were 0.962 ± 0.095 and 79.52 ± 14.02, respectively. Comparing with poor CVH participants (EQ-5D-5L utility scores and VAS scores: 0.954 ± 0.111 and 78.44 ± 14.29), people with intermediate and ideal CVH had higher EQ-5D-5L utility scores (0.969 ± 0.079 and 0.959 ± 0.099) and VAS scores (80.43 ± 13.65 and 79.28 ± 14.14). ICH scores were positively correlated with EQ-5D-5L utility scores (0.007 (0.004, 0.009)) and VAS scores (0.295 (0.143, 0.446)), respectively.

**Conclusions:** Higher ICH scores is positive associated with better HRQoL in rural population, which suggests that improvement of cardiovascular health may help to enhance HRQoL among rural population.

## Introduction

Cardiovascular disease (CVD) is now the leading cause of premature mortality and disability in world [1, 2]. In 2010, ideal cardiovascular health (ICH) was formulated by the American Heart Association to reduce deaths from all CVDs [3]. ICH was defined with four ideal health behaviors (ideal smoking status, ideal body mass index (BMI), ideal physical activity, and ideal diet) and four ideal health factors (ideal smoking status, ideal total cholesterol (TC), ideal blood pressure (BP), and ideal fasting plasma glucose (FPG)) [3]. Prospective studies consistently indicated that individuals with a higher number of ideal cardiovascular health metrics have lower risks of hypertension [4], type 2 diabetes mellitus [5], CVD events [6, 7], and all-cause mortality [4].

Health-related quality of life (HRQoL) refers to the individual’s health status under the influence of illness and injury, medical intervention, aging and social environment change, as well as the subjective satisfaction associated with its economic, cultural background and value orientation. It is a multidimensional concept which was main contented by health status and subjective satisfaction [8]. The European Quality of Life Five Dimension Five Level Scale (EQ-5D-5L) is one of the simplest and most commonly used self-reported instruments to evaluate HRQoL due to its simple, low response requirements, and generally high acceptance [9].

Prior literature has demonstrated an association of individual cardiovascular risk factors with HRQoL [10, 11]. However, most studies considered single cardiovascular risk factors. Although some previous studies also have reported the association between cardiovascular health and HRQoL [12–14]. These studies were conducted in western countries and used the Medical Outcomes Study 12-Item Short Form Health Survey (SF-12) and HRQoL-4 tool. Hence, the aim of this study was to explore the association between ICH and HRQoL using EQ-5D-5L in Chinese rural population.

## Methods

### Study Design and Participants

The Henan Rural Cohort Study is a prospective study of chronic non-communicable diseases among a large sample of rural people established in Henan Province, China, from 2015 to 2017. In short, the cohort recruited participants aged from 18 to 79 by multi-stage stratified cluster sampling. 39,259 people were included in the cohort study, with a response rate of 93.7%. The details of this cohort have been described elsewhere [15]. In this study, 23,510 participants with complete information on EQ-5D-5L were included. Then, the participants were further excluded if they [1]: were diagnosed with coronary heart disease (n = 1,247) [2]; were diagnosed with stroke (n = 1710) [3]; were missed information needed in the present study (n = 78). Finally, 20,683 adults were ultimately included in the current study.

This study was consistent with “Zhengzhou University Life Science Ethics Committee” [Ethics approval code [2015] MEC (S128)]. Participants were required to provide informed consent.

### Data Collection

A standard questionnaire was administered by face to face interviews, including demographic characteristics (age, gender, educational level, marital status, and per capita monthly income), lifestyles (smoking, drinking, and physical activity), behaviors, dietary patterns (through Food Frequency Questionnaire (FFQ)), individual history of diseases and medication use were collected. The International Physical Activity Questionnaire (IPAQ 2001) was used to assess the levels of physical activity (16). The FFQ based on the Dietary Guidelines for Chinese Residents and the eating habits of Henan people. Previous studies have shown that the FFQ has good reproducibility and validity (17). Individual histories of disease and medication were self-reported by the participants. The height and weight of participants were measured twice, and the average readings were computed to analyze. Body mass index (BMI, kg/m^2^) was calculated as weight (kg) divided the square of height (m). Blood pressure was measured three times by electronic sphygmomanometer in the right arm in a sitting position after at least 5 min rest. There were 30s intervals between the three measurements. Venous blood samples were collected from subjects after an overnight fast of at least 8 hours and stored in −80°C cryogenic refrigerator before analysis. The FBG and TC were measured by Roche Cobas C501 automatic biochemical analyzer. The details of the equipment for anthropometric and clinical examinations have been introduced elsewhere (15).

### Ideal Cardiovascular Health Metrics

ICH metrics were as following according to the AHA (3) : ideal smoking status, never a smoker; ideal BMI, BMI <25 kg/m^2^; ideal physical activity, physical activity ≥150 min/wk of moderate intensity or ≥75 min/wk of vigorous intensity or ≥150 min/wk of moderate-vigorous intensity combination; ideal diet, ≥ 4 components; ideal TC, TC < 5.18 mmol/L untreated; ideal BP, SBP <120/DBP< 80 mm Hg untreated, and ideal FPG, FPG < 5.6 mmol/L untreated. (3) Furthermore, ICH was defined as follows: the simultaneous presence of 4 IHB (ideal smoking status, ideal BMI, ideal PA, and ideal diet) and 4 IHF (ideal smoking status, ideal TC, ideal BP, and ideal FPG) in the absence of a history of cardiovascular disease (3). We made some adaptations as appropriate for the healthy diet score. Ideal diet was defined as healthy diet score ≥4 components (18), including fruits and vegetables ≥ 500 g/d, fish ≥ 200 g/week, soybean products ≥ 125 g/d, red meat < 75 g/d, and drinking tea.

### EQ-5D-5L

In this study, the EQ-5D-5L was utilized to evaluate the HRQoL of participants. The five dimensions of EQ-5D-5L were consisted of mobility (MO), self-care (SC), usual activities (UA), pain/discomfort (PD) and anxiety/depression (AD). Each dimension had five levers including no problems, slight problems, moderate problems, severe problems and extreme problems. For example, participants were asked if they had any problems walking. If they did, they were asked if it was serious and if they did not, they were asked if it was a slight or a moderate problem. Participants that reported their problems as serious were asked if they could walk at that moment. If they could, they were classified as having severe problems walking and if they could not, they were classified as having extreme problems.

The EQ-5D utility scores were calculated based on the recently available Chinese value set for the EQ-5D-5L instrument [19]. Utility = 1 − MO × Ln − SC × Ln − UA × Ln − PD × Ln − AD × Ln (n = 1, 2, 3, 4, 5). Scores ranged from −0.200 to 1.000, with one representing full health, 0 representing death, and a score <0 representing a health status worse than death. The EQ-5D-5L also included a visual analogue scale (VAS) where participants marked their own health on a scale ranging from “best imaginable” (100) to “worst imaginable” state of health (0).

### Statistical Analysis

The continuous data were represented by mean and standard deviation (SD) and the categorical data were tabulated with frequencies and percentages. Student's t test and chi-square test was used to compare the difference between groups.

Due to the distribution of the EQ-5D utility was skewed and the utility score was censored at 1, multivariate Tobit regression model was performed to assess the association between ICH and EQ-5D utility scores [20]. A generalized linear model (GLM) was chosen assess the association between ICH and VAS scores, because the VAS score was abnormal distribution continuous variable. Apart from the linear regression model, we also used a logistic regression model to examine the association between EQ-5D utility scores and VAS scores and the prevalence of ideal CVH (ICH scores ≥5). A range of potential confounders were adjusted, including age (<40, 40–60 or ≥60 years), gender (men or women), educational level (primary school or illiteracy, junior high school, or high school or above), income (<500, 500–100 or ≥1000 RMB per month) and drinking (no drinking or current drinking). Results were expressed as increased ICH scores and 95% confidence intervals (95% CIs) or odds ratio of ideal CVH associated with increment of EQ-5D utility scores and VAS scores. The potential modification effects of gender, age, education level, income and drinking were examined by adding an interaction term into the adjusted model. All statistical analyses were performed by SPSS software V.21.0, STATA 15 for Windows and R version 3.6.3.

## Results

### Characteristics of the Participants

A summary of participants’ demographic characteristics is shown in Table 1. Of all participants, 6,825 (33.00%) showed Ideal CVH (ICH scores ≥ 5). Lower mean age and BMI, higher fractions of women and married/cohabiting were observed among those with ideal ICH than others. Participants with ideal CVH tended to be non-smoker and non-drinkers. In different ICH groups, the EQ-5D utility scores and VAS scores of participants with intermediate and ideal CVH were higher than those poor CVH, indicating that participants with ideal CVH had higher HRQoL.

**TABLE 1:** Characteristics of the participants (Collected during 2015–2017, China).

Variables	Total	Poor CVH	Intermediate CVH	Ideal CVH	*p*
Age (years, mean ± SD)	54.26 ± 12.75	56.91 ± 11.95	56.28 ± 11.77	49.84 ± 13.43	<0.001
Sex					<0.001
Men	8,335 (40.30)	2,149 (62.15)	4,381 (42.04)	1805 (26.45)	
Women	12,348 (59.70)	1,289 (37.49)	6,039 (57.96)	5,020 (73.55)	
Marital status					0.060
Married/cohabiting	18,753 (90.67)	3,127 (90.95)	9,399 (90.20)	6,227 (91.24)	
Unmarried/divorced/widowed	1930 (9.33)	311 (9.05)	1,021 (9.80)	598 (8.76)	
Education level					<0.001
Primary school or illiteracy	8,496 (41.08)	1,383 (40.23)	4,591 (40.06)	2,522 (36.95)	
Junior high school	8,085 (39.09)	1,322 (38.45)	3,996 (38.35)	2,767 (40.54)	
High school or above	4,102 (19.83)	733 (21.32)	1833 (17.59)	1,536 (22.51)	
Income (RMB per month)					<0.001
<500	7,433 (35.94)	1,148 (33.39)	3,891 (37.34)	2,394 (35.08)	
500∼	6,599 (31.9)	1,150 (33.45)	3,378 (32.42)	2071 (30.34)	
1,000∼	6,651 (32.16)	1,140 (33.16)	3,151 (30.24)	2,360 (34.58)	
Smoking					<0.001
No smoking	14,965 (72.35)	1,580 (45.96)	7,423 (71.24)	5,962 (87.36)	
Current smoking	5,718 (27.65)	1858 (54.04)	2,997 (28.76)	863 (12.64)	
Drinking					<0.001
No drinking	16,004 (77.38)	2061 (59.95)	8,006 (76.83)	5,937 (86.99)	
Current drinking	4,679 (22.62)	1,377 (40.05)	2,414 (23.17)	869 (13.01)	
BMI (kg/m^2^ mean ± SD)	24.93 ± 3.60	27.60 ± 3.13	25.44 ± 3.46	22.82 ± 2.74	<0.001
Utility scores, mean (SD)	0.962 ± 0.095	0.954 ± 0.111	0.959 ± 0.099	0.969 ± 0.079	<0.001
VAS scores, mean (SD)	79.52 ± 14.02	78.44 ± 14.29	79.28 ± 14.14	80.43 ± 13.65	<0.001
Total	20,863 (100.00)	3,438 (16.62)	10,420 (50.38)	6,825 (33.00)	

SD, standard deviation; BMI, body mass index; EQ-5D-5L utility scores: The European Quality of Life Five Dimension Five Level Scale utility scores; VAS scores: Visual analogue scale scores; Poor CVH, poor cardiovascular health (0–2 of ideal cardiovascular health scores); Intermediate CVH, intermediate cardiovascular health (three to four of ideal cardiovascular health scores); Ideal CVH, ideal cardiovascular health (five to seven of ideal cardiovascular health scores).

### Reported Health Problems

The percentage of respondents who reported problems (no, slight, moderate, severe, or extreme problems) based on the EQ-5D-5L questionnaire is shown in Table 2. The dimension with the highest proportion of patients with self-reported problems was the pain/discomfort dimension (4.23%), followed by mobility dimension (2.50%). The self-care dimension of was the least report (0.91%). In these three dimensions (mobility, self-care and usual activities), the proportion of participants with ideal CVH with problems was lower than that of participants with non-ideal CVH. Except for the pain/discomfort dimensions, other four dimensions were all significantly different among ICH groups (all *p* < 0.001).

**TABLE 2:** The association between ideal cardiovascular health metrics and health-related quality of life (Collected during 2015–2017, China).

ICH metrics	Total, n (%)	EQ-5D-5L utility scores^a^			VAS scores^b^		
(ref. = non-ideal)		COE	SE	*p*	*β*	SE	*p*
Total cholesterol							
Ideal	12,451 (60.20)	0.012	0.004	0.004	0.447	0.199	0.024
Blood pressure							
Ideal	7,976 (38.56)	0.002	0.004	0.673	0.017	0.206	0.933
Fasting plasma glucose							
Ideal	14,173 (68.52)	0.010	0.004	0.016	0.597	0.208	0.004
Smoking							
Ideal	14,965 (72.35)	−0.012	0.007	0.072	0.347	0.326	0.286
Physical activity							
Ideal	18,602 (89.94)	0.046	0.007	0.001	1.973	0.321	<0.001
BMI (kg/m^2^)							
Ideal	10,923 (52.81)	0.007	0.004	0.079	0.039	0.191	0.840
Healthy diet score							
Ideal	105 (0.51)	−0.010	0.029	0.734	0.363	1.343	0.787

ICH, Ideal cardiovascular health; BMI, body mass index; EQ-5D-5L utility scores: The European Quality of Life Five Dimension Five Level Scale utility scores; VAS scores: Visual analogue scale scores.

^a^ Multivariate Tobit regression model.

^b^ Generalized linear model.

### Associations Between Ideal Cardiovascular Health and Health-Related Quality of Life

**FIGURE 1:**
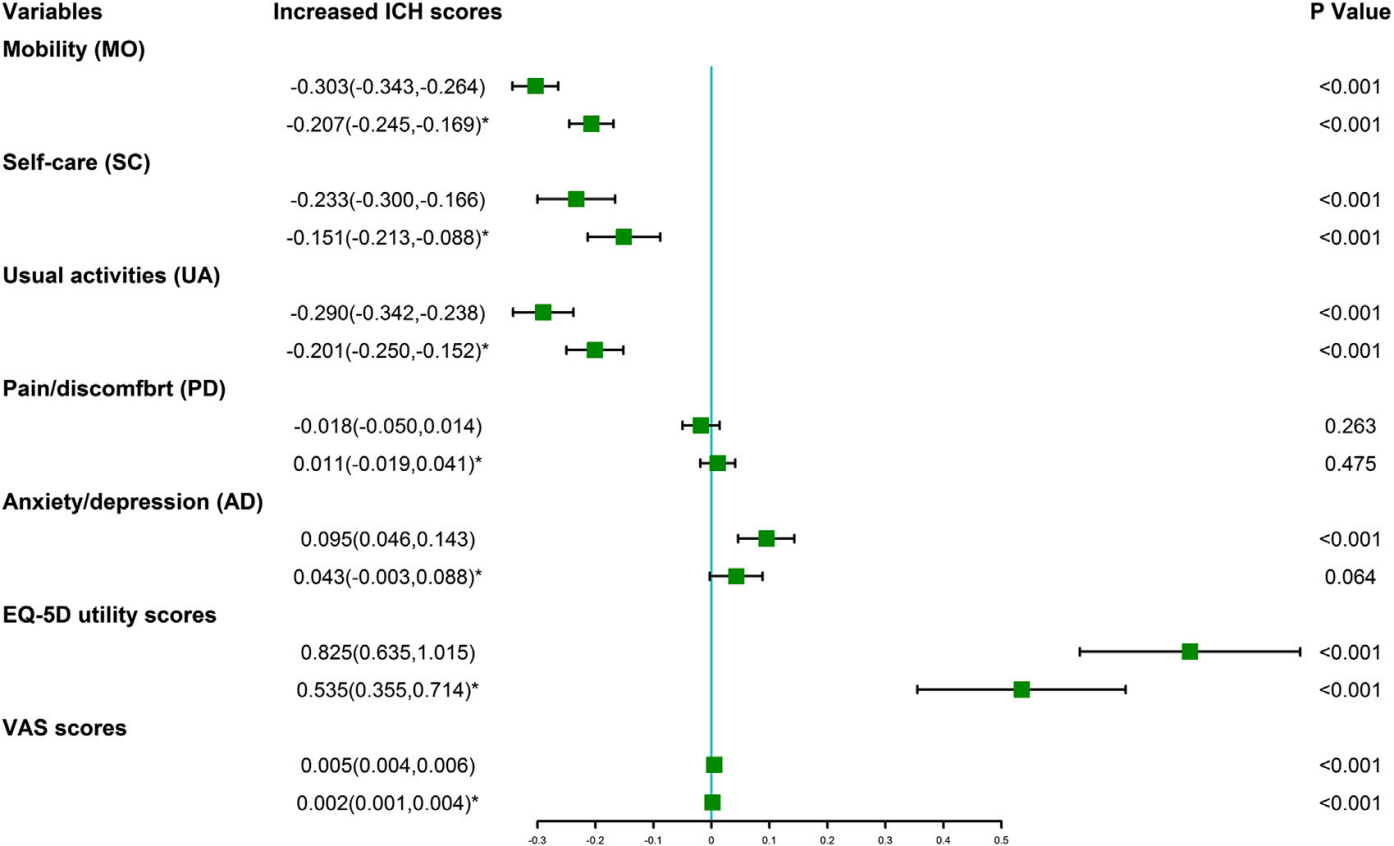
Increased ideal cardiovascular health scores (and 95%CI) associated with the European quality of life five dimension five level scale utility scores and visual analogue scale scores (Collected during 2015–2017, China). *adjusted model, potential confounders were adjusted, including age, gender, educational level, income and drinking.

**FIGURE 2:**
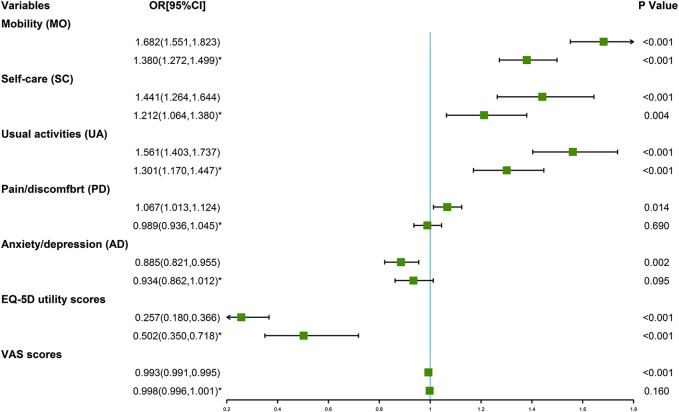
Odds ratio of ideal cardiovascular health (and 95% CI) associated with the European quality of life five dimension five level scale utility scores and visual analogue scale scores (Collected during 2015–2017, China). *adjusted model, potential confounders were adjusted, including age, gender, educational level, income and drinking.

Table 3 shows the association between ICH metrics and HRQoL. Ideal total cholesterol, ideal fasting plasma glucose and ideal physical activity were positively related with EQ-5D utility scores and VAS scores, respectively. In addition, IHB scores, IHF scores and ICH scores were positively related with EQ-5D utility scores and VAS scores, respectively (Supplementary Table S1). The results of crude linear regression models showed higher ICH scores was associated with increment of EQ-5D utility scores and VAS scores. After adjusted for potential confounders, the ICH scores increased associated with increment of EQ-5D utility scores and VAS scores were 0.535 (0.355, 0.714) and 0.002 (0.001, 0.004), respectively (Figure 1). The results of adjusted logistic regression models showed that the odds ratios and 95% CIs of ideal ICH associated with increment of EQ-5D utility scores and VAS scores were 0.502 (0.350, 0.718) and 0.998 (0.996, 1.001), respectively (Figure 2).

**TABLE 3:** The association between ideal cardiovascular health scores and health-related quality of life (Collected during 2015–2017, China).

Ideal scores	Total, n (%)	Utility scores^a^			VAS scores^b^		
		Mean ± SD	COE (95%CI)	*p*	Mean ± SD	*β* (95%CI)	*p*
IHB scores			0.012 (0.016,0.018)	<0.001		0.519 (0.307, 0.892)	<0.001
0	422 (2.04)	0.966 ± 0.097			79.06 ± 13.47		
1	3,063 (14.81)	0.961 ± 0.108			79.44 ± 14.20		
2	10,087 (48.77)	0.960 ± 0.097			79.50 ± 14.07		
3	7,086 (34.26)	0.964 ± 0.087			79.61 ± 13.92		
4	25 (0.12)	0.956 ± 0.070			78.52 ± 13.84		
IHF scores			0.005 (0.001, 0.009)	0.023		0.282 (0.088, 0.476)	0.004
0	729 (3.25)	0.968 ± 0.088			79.56 ± 13.50		
1	3,684 (17.81)	0.955 ± 0.106			78.34 ± 14.30		
2	6,530 (31.57)	0.957 ± 0.102			78.89 ± 14.22		
3	6,139 (39.68)	0.963 ± 0.932			79.91 ± 14.02		
4	3,601 (17.41)	0.973 ± 0.071			81.19 ± 13.28		
ICH scores			0.007 (0.004, 0.010)	<0.001		0.295 (0.143, 0.446)	<0.001
0	90 (0.44)	0.970 ± 0.072			79.27 ± 13.06		
1	709 (3.43)	0.951 ± 0.128			78.67 ± 13.82		
2	2,639 (12.76)	0.954 ± 0.108			78.35 ± 14.45		
3	4,914 (23.76)	0.956 ± 0.107			78.81 ± 14.22		
4	5,506 (26.62)	0.961 ± 0.090			79.71 ± 14.06		
5	4,518 (21.84)	0.967 ± 0.084			80.15 ± 13.75		
6	2,297 (11.11)	0.9726 ± 0.068			80.96 ± 13.44		
7	10 (0.05)	0.9733 ± 0.045			79.80 ± 13.84		
ICH group							
Ideal CVH	6,825 (33.00)	0.969 ± 0.079	0		80.43 ± 13.65	1	
Intermediate CVH	10,420 (50.38)	0.959 ± 0.099	0.016 (0.005, 0.027)	0.004	79.28 ± 14.14	1.110 (0.574, 1.646)	<0.001
Poor CVH	3,438 (16.62)	0.954 ± 0.111	0.027 (0.015, 0.040)	<0.001	78.44 ± 14.29	1.193 (0.600, 1.787)	<0.001

Utility scores: The European Quality of Life Five Dimension Five Level Scale utility scores; VAS scores: Visual analogue scale scores; IHB scores, Ideal health behaviors scores; IHF scores, Ideal health factors cores; ICH scores, ideal cardiovascular health scores; Poor CVH, poor cardiovascular health (0–2 of ideal cardiovascular health scores); Intermediate CVH, intermediate cardiovascular health (three to four of ideal cardiovascular health scores); Ideal CVH, ideal cardiovascular health (five to seven of ideal cardiovascular health scores).

^a^ Multivariate Tobit regression model.

^b^ Generalized linear model.

### Interaction Analyses of Ideal Cardiovascular Health and Health-Related Quality of Life

In the interaction analyses, it revealed that the association between ICH and HRQoL was modified by age, gender, income and educational lever and drinking in Supplementary Table S2. Stronger associations between ICH and EQ-5D utility scores were presented among women participants, those aged ≥60 years, with low income, low education level and non-drinker participants. However, there was an significantly positive correlation between ICH and VAS scores, and stronger associations between ICH and VAS scores were presented among men, those aged <40 years, with low income, high education level and drinker.

## Discussion

To the best of our knowledge, this is the first study to examine the effect of HRQoL on ideal cardiovascular health (ICH) use the European Quality of Life Five Dimension Five Level Scale (EQ-5D-5L) among rural adults. Based on the baseline survey of the Henan Rural Cohort, participants’ cardiovascular health was evaluated via ICH scores and their HRQoL were estimated by EQ-5D-5L. The EQ-5D utility scores and VAS scores of participants with intermediate and ideal CVH were higher than those poor CVH. ICH scores were positively correlated with EQ-5D utility scores and VAS scores, respectively. Stronger associations between ICH and EQ-5D utility scores were presented among women participants, those aged ≥60 years, low income, low education level and non-drinker. However, stronger associations between ICH and VAS scores were presented among men, those aged <40 years, low income, high education level and drinker. In addition, we further conclude that HRQoL was the influencing factor of ideal cardiovascular health.

Prospective studies consistently demonstrated that individuals with a higher number of ideal cardiovascular health metrics have lower risks of hypertension [4], type 2 diabetes mellitus [5], CVD events (stroke, heart failure, myocardial infarction, and fatal coronary disease) [6, 7], and all-cause mortality [4]. Our results extend these findings providing evidence by demonstrating the possible beneficial effect of cardiovascular health on HRQoL. People with cardiovascular conditions or diabetes had higher risk of reporting poor HRQoL outcomes [21]. A greater number of multiple CVD risk factors may be associated with more detrimental impairment of HRQoL [22]. A recent study from the National Health and Nutrition Examination Survey, showed that compared to those with poor CVH, individuals in intermediate and ideal CVH were 44 and 71% less likely to report being in fair/poor health [12]. Another study of the Coronary Artery Risk Development in Young Adults study reported that t maintaining ideal CVH from early adulthood results in higher health-related quality of life in middle age [13].

HRQoL was considered both a risk factor for and consequence of low levels of CVH [23]. Previous studies have conceptualized lower self-rated health as a risk factor for low CVH [12, 14]. Similar results were found in our study, which was illustrated that EQ-5D utility scores and VAS scores were associated with increased ICH scores and higher risk of ideal CVH. Our study further found that ideal health behaviors and ideal health factors were positively correlated with EQ-5D utility scores and VAS scores, respectively. A review showed cross-sectional data showed a consistently positive association between physical activity level and health-related quality of life [11]. Physical activity of recommending levels were associated with better HRQoL [24]. A study reaffirms the significant association between smoking and HRQoL in a large nationally representative sample [25]. Adults with the metabolic syndrome experience worse health-related quality of life than adults without this syndrome [10]. Patients with longer duration of hypertension scored lower on HRQoL than others [26]. Dyslipidaemia was correlated with a lower HRQoL [27]. Thus, health behaviors and health factors included in the definition of overall cardiovascular health represents an important step toward to improve the HRQoL status, and it is urgent to promote status on smoking, diet and blood pressure.

In the interaction analyses, it revealed that the association between ICH and HRQoL was modified by age, gender, income and educational level and drinking. Stronger effects of ICH on HRQoL were observed among those aged ≥60 years, low income and non-drinking. For different ages, genders and different social factors, our findings make us think about possible interventions to improve HRQoL. The oldest old population has become the fastest growing segment who have excess need of care and social support. Quality of life was not only associated with age-related diseases, but also correlated with a range of health-related lifestyles, and factors indicating social and family support [28]. it is crucial to improve the health-related quality of life (HRQoL) of these populations. Previous study reported the relationship between socio-economic status and health [29]. As the largest developing country, China is experiencing rapidly growing income inequality. To improve people's quality of life, it is important to ameliorate their socio-economic status, especially in rural area. Drinking habits showed lower physical health-related quality of life scores [30]. There was a negative correlation between alcohol consumption and health related quality of life [31]. Thus, limiting alcohol consumption may increase the correlation between cardiovascular health and health-related quality of life.

### Strengths and Limitations

Our research has some advantages. Firstly, compared with the previous research [12, 14], we used EQ-5D-5L for the first time to evaluate the association between ideal cardiovascular health and health-related quality of life of Chinese rural population, bringing less ceiling effect [9]. secondly, we applied the newly developed EQ-5D-5L value set [19] based on Chinese population to Chinese rural population for the first time, avoiding bias due to cultural and population discrepancies compared with the value set based on other countries. In addition, most of the research objects are middle-aged and elderly people, which can better represent the current structure of Chinese rural population. Therefore, our findings can be extended to all rural areas in China. Nevertheless, several limitations should also be considered. Firstly, this is a cross-sectional study, thus do not accurately describe causality. Secondly, some residents, such as college students and migrant workers, were not included in the scope of this study because they were studying or working outside. These people are more likely to be young and healthy and have a higher prevalence of ICH, which may lead to the underestimation of ICH in the rural population. Finally, the EQ-5D-5L value set for China was developed from urban areas rather than rural areas, which may introduce bias to our study.

## Conclusion

In rural China, achieving better cardiovascular health metrics is associated with better HRQoL, which may extend the benefits of improving cardiovascular health beyond reducing the incidence of CVD and disability. Improvement of cardiovascular health may help to improve HRQoL among rural population of China.

## Data Availability

The raw data supporting the conclusions of this article will be made available by the authors, without undue reservation.
